# Spatio-temporal dynamics and drivers of cropland ecosystem services value during the past two decades in Yangtze river economic belt, China

**DOI:** 10.3389/fpubh.2025.1622093

**Published:** 2025-06-27

**Authors:** Hanjun Xia, Pengbo Zhang, Qi Zhang, Baofa Peng, Chenxin Zhou, Qian Zhu, Songchao Chen, Bifeng Hu

**Affiliations:** ^1^College of Geographical Sciences and Tourism, Hunan University of Arts and Science, Changde, Hunan, China; ^2^School of Economic Geography, Hunan University of Finance and Economics, Changsha, China; ^3^Department of Land Resource Management, School of Public Administration, Jiangxi University of Finance and Economics, Nanchang, China; ^4^College of Environmental and Resource Sciences, Zhejiang University, Hangzhou, China

**Keywords:** Spatio-temporal dynamics, cropland ecosystem services value, multi-scale geographically weighted regression model, driving factors, Yangtze river economic belt

## Abstract

**Introduction:**

Cropland ecosystem is one of the fundamental natural resources for human survival and development, serving not only as the core carrier of food production but also as an important provider of ecological services. Clarifying the spatio-temporal variation of the cropland ecosystem service value (Crop-ESV) and understanding its main drivers are critical for maintaining and regulating cropland ecosystem functions.

**Methods:**

Thus, this study systematically assessed the Crop-ESV in the Yangtze River Economic Belt (YREB) in China and mapped it at 1 km spatial resolution from 2001 to 2020. Secondly, the Sen-MK trend analysis was used to analyze the change trend of Crop-ESV in the YREB. Finally, structural equation modeling and multi-scale geographically weighted regression were employed to analyze the influence of physical and socio-economic factors on the Crop-ESV within the YREB.

**Results:**

Our results showed that the Crop-ESV in the YREB exhibited an increasing trend over the past two decades, from 10,674 billion yuan in 2001 to 11,564 billion yuan in 2020, representing an average annual increase of 0.94 million yuan/ha (*p*-value < 0.05). Moreover, the Crop-ESV showed significant spatial heterogeneity, with hot spots predominantly clustered in the west, while cold spots were primarily located in the east. Terrain was identified as the primary positive driver of Crop-ESV, whereas meteorological conditions were the main negative driver.

**Discussion:**

These findings contribute to safeguarding food security and ecological integrity in both the YREB and China, and provide a valuable reference for harmonizing development and optimizing policies within the YREB and similar regions.

## 1 Introduction

Cropland ecosystem is a complex system that contains interactions between natural and anthropogenic factors (1). Cropland ecosystem is the foundation of human living and growth (2). It not only provides 66% of global food output (3), but also has indirect service capabilities such as gas regulation, water conservation and soil conservation, rendering it one of the most prominent ecosystems in the world (4). Nevertheless, rapid socio-economic and population growth has damaged the ecosystem environment and negatively impacted the cropland ecosystem service value (Crop-ESV) (5). In-depth research and quantitative assessment of the Crop-ESV and understanding of its spatio-temporal evolution patterns will help to scientifically recognize the ecological benefits of cropland, promote the construction of high-standard basic cropland, and advance the green and sustainable agriculture development (6, 7).

There has been a considerable amount of research focusing on the valuation of ecosystem services, with most studies focusing on other land use types such as: forests, sandy land, water, and grasslands (8). For example, Cai et al. (9) adopted a variety of methods to assess the Crop-ESV in Qingdao city. Wang et al. (10) implemented valuation method to calculate the Crop-ESV to estimate the changes in ecosystem services because of municipalization in Guangdong, Hong Kong and Macao. Liu et al. (11) applied meta-regression analysis to synthesize the ecosystem service value of grassland and the affecting factors. Few studies have focused on the Crop-ESV. Liu et al. (12) employed multi-scale trends to analyze the trends in four ecosystem services value as well as their factors in Hongshanda Sandland, Inner Mongolia. Zhang et al. (13) analyzed spatio-temporal variations and drivers of ESV in Danjiangkou Reservoir and its upstream zone. Wang et al. (14) applied a comprehensive assessment of ESV in mangrove forests in Guangxi Province based on field surveys and remote sensing data. However, most of current studies mainly focused on small spatial scale and they usually reveal a strong spatio-temporal change of Crop-ESV. Therefore, stronger spatio-temporal variation of Crop-ESV is expected in larger spatial scale because of the complex impacts of various natural and anthropogenic factors.

The Crop-ESV is influenced by a variety of factors such as meteorology, soil properties, terrain and anthropogenic activities (15, 16). Most current studies used spatial autocorrelation analysis or Geo-detector to explore the main drivers of Crop-ESV. Among which, Li et al. (17) assessed spatial autocorrelation between land use types and ecosystem service values using global Moran’s I and local Moran’s I. Peng et al. (18) examined the drivers of global ecosystem services through Geo-detector. Yang et al. (19) used Geo-detector to study the drivers of ecosystem service values in tropical rainforests. However, these methods lack parametric statistical inference and often fail to fully explore the causal relationships and relative contributions between variables (20). Structural Equation Modeling (SEM) is a statistically based multivariate analysis method for testing and estimating complex relationships between variables (21). It integrates factor analysis and path analysis to not only identify spatial effects, but also to quantify and predict relationships between variables (22). What’s more, Multiscale Geographically Weighted Regression (MGWR) is an improved geo-spatial regression method. It is able to handle both spatial heterogeneity and localized effects, and is suitable for scenarios where variable relationships vary spatially (23, 24). Consequently, the SEM and MGWR provide more ideal tools to identify the main factors of spatio-temporal change of Crop-ESV (8, 25–28).

Although there have been some studies on the ecosystem service value in the YREB. For example, Tu et al. investigated the coupling coordination relationship and spatio-temporal heterogeneity between ecosystem services and new-type urbanization (29). Qu et al. analyzed the spatial relationships between carbon emission efficiency and total ecosystem services and the spatial spillover effects of carbon emission efficiency in the YREB from 1990 to 2020 (30). Qu et al. discussed the relationship between urbanization and the ecosystem service scarcity value in the YREB (31). Wu et al. assessed the combined effects of climate and land use on water-related ecosystem services in the YREB (32). Yao et al. examined the dynamic impact of landscape pattern on the interactions between ecosystem service trade-offs in the YREB from 1990 to 2020 (33). Most of the studies focus on the coupling relationship of ecosystem services, but few studies pay attention to the basic characteristics and distribution pattern of the Crop-ESV in the YREB, and investigate the influencing factors of the Crop-ESV (7, 34, 35). The YREB is a critical region for China’s socioeconomic development, food production, and ecological protection (16), and six of China’s nine major grain-producing regions are located in the YREB (7, 36). Enhancing the understanding of the spatio-temporal distribution of Crop-ESV in the YREB and their influencing factors is essential for improving cropland quality, constructing high-standard cropland, and ensuring national food security. Furthermore, numerous studies have demonstrated the relevance of ecosystem service values to the 17 Sustainable Development Goals (SDGs) set by the United Nations, with food production and water conservation, habitat and biodiversity conservation, and carbon storage and sequestration all recognized as contributing to >14 SDG targets (37, 38).

Hence, in this study, we mapped the spatio-temporal pattern of Crop-ESV in the YREB from 2001 to 2020, understood the distribution of hot and cold spots of Crop-ESV in the YREB in the last two decades, and figured out the driving factors affecting the Crop-ESV in the YREB. Our research can provide useful guidance to improve the cropland quality, build high-standard cropland, guarantee national food security, but also contribute China’s solutions to achieving the SDGs (39–42).

## 2 Materials and methods

### 2.1 Study area

**Figure 1:**
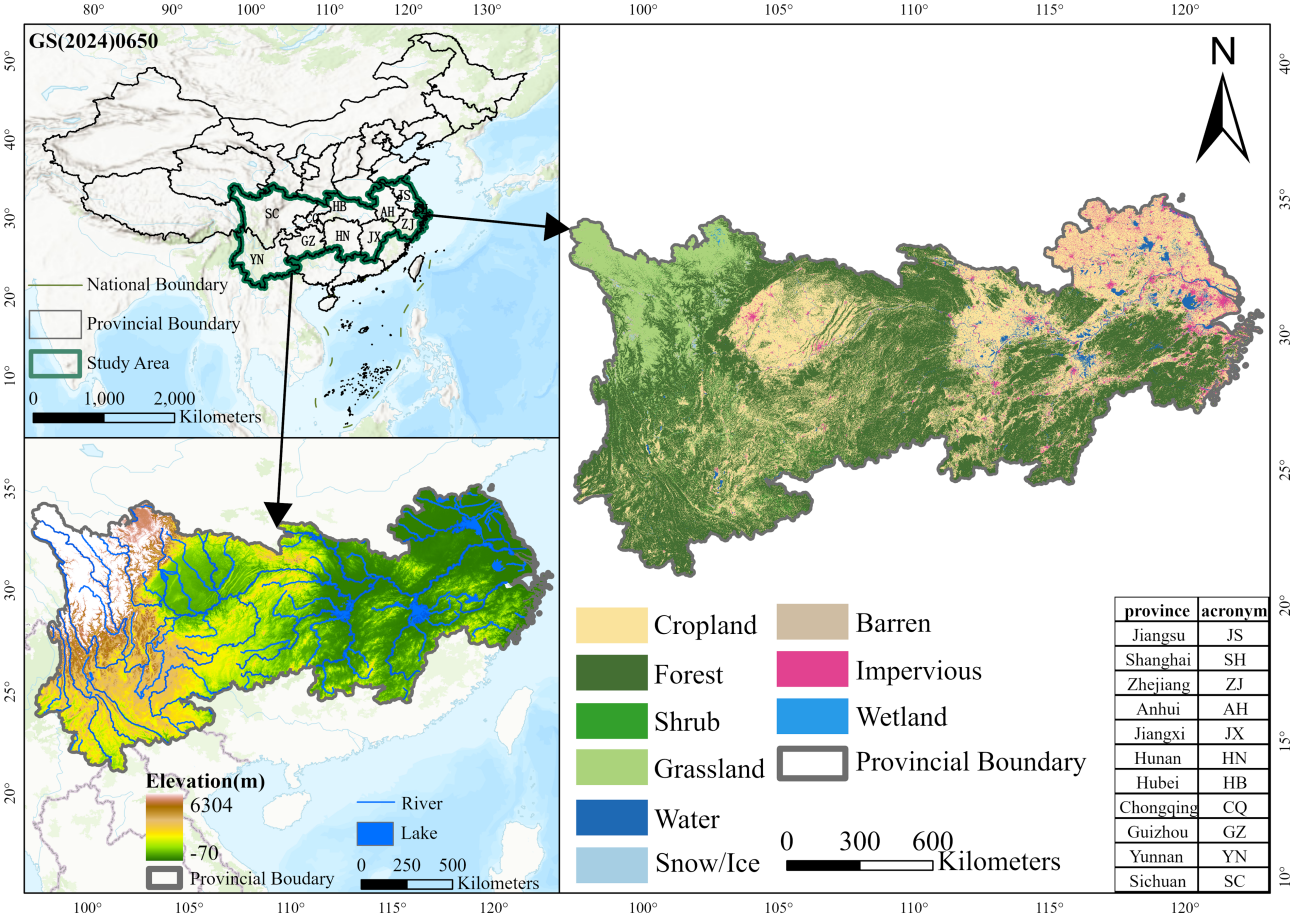
Study area.

The Yangtze River Economic Belt (YREB) covers 11 provinces and municipalities, including Shanghai, Jiangsu, Zhejiang, Anhui, Jiangxi, Hubei, Hunan, Chongqing, Sichuan, Guizhou and Yunnan, with an area of about 2,052,300 square kilometers, accounting for 21.4% of the area of China (43). The YREB has a subtropical monsoon climate and encompasses 279,600 km^2^ of cropland, making it a critical food production area in China (44). According to the division of upstream, midstream and downstream, the downstream regions include four provinces and cities of Jiangsu, Shanghai, Zhejiang and Anhui; the midstream regions include three provinces of Jiangxi, Hunan and Hubei; and the upstream regions include four provinces and cities of Chongqing, Guizhou, Yunnan and Sichuan (Figure 1).

### 2.2 Calculation of crop-ESV

This study was conducted to systematically account for the Crop-ESV in the YREB from 2001 to 2020. The four core services most closely linked to human life, food production (FP), gas regulation (GR), water conservation (WC) and soil conservation (SC), were selected, and a hybrid method combining multi-source remote sensing and ground observation was used for sub-measurement. Firstly, based on the dynamic crop yield monitoring data, the market price method was used to quantify the annual economic contribution of FP (45); the GR value covered the dual paths of carbon dioxide fixation and oxygen release (46), which were monetized with reference to the Nordic carbon tax standard and the cost of industrial oxygen production, respectively (47); the WC value was estimated by integrating the water balance model and the shadow engineering method, and the ecological benefits are discounted by the construction cost per unit of reservoir capacity (48, 49); and the SC value consisted of sediment reduction and nutrient retention, with the former using the engineering replacement cost method and the latter combining the market price of elements and soil remediation costs (50). The final Crop-ESV was determined by summing the four service values (The details for calculation of crop-ESV are included in Supplementary material).

### 2.3 Sen-MK trend analysis

The Sen-MK trend assessment is a nonparametric statistical method combined Sen slope estimator and Mann-Kendall test (51). It is widely used in environmental sciences (52), hydrology (53), and meteorology (54) for detecting trends and their significance in time series data. In this paper, the significant level is set to 0.05, so when |Z| is greater than 1.96, there is a significant trend of change in Crop-ESV.

### 2.4 Hot spot analysis

In our research, hot spot analysis was applied to revealing the spatial clustering pattern and temporal evolution characteristics of Crop-ESV. Based on the Getis-Ord Gi* spatial statistical model (55), statistically significant Crop-ESV clustering areas were accurately identified by calculating the standardized Z scores of each spatial unit. Among them, hotspot zones characterized the concentrated distribution zones of high-value clusters of Crop-ESV, while cold spot zones reflected the clustering space of low-value elements.

### 2.5 Structural equation model

In this study, structural equation modeling (SEM) is used to analyze the mechanism of multivariate effects (56). It effectively explains the complex causal network between the variables by integrating factor analysis and path analysis (21). To characterize the spatio-temporal variability of the Crop-ESV in the YREB, this study focuses on analyzing the impact intensity of each driver on Crop-ESV as well as the impact mechanisms in 2020, so as to reveal the driving pathways and interactive impacts of physical and human factors on Crop-ESV.

### 2.6 Multiscale geographically weighted regression

Multiscale geographically weighted regression (MGWR) resolves the multiscale spatial effects of variables through differential bandwidth settings (23). Compared to traditional GWR, MGWR allows each variable to autonomously optimize the level of spatial smoothing, thus enhancing the heterogeneity modeling accuracy (57). In this study, MGWR 2.2 was used for modeling based on 26 driving factors. Adaptive bisquare was used in the model construction to capture spatially localized features, and the bandwidth was optimized with the AICc criterion, and the optimal bandwidth was determined by adjusting the bandwidth of each explanatory variable to minimize the AICc value. All variables were Z-score normalized to ensure parameter comparability, ultimately demonstrating that MGWR significantly outperforms conventional models through an independent bandwidth optimization mechanism.

### 2.7 Data collection

The data used in our research cover land use data, natural and socio-economic data. The land use data was obtained from the Chinese Land Cover Dataset (CLCD) for the period of 2001–2020 (58). The CLCD data was divided into the following seven categories: cropland, forest, shrubland, grassland, barren land, water, and impervious surfaces. At first, we projected the CLCD dataset to WGS 1984 UTM Zone 49 N. Because the spatial resolution for CLCD is 30 m, the nearest method was applied to resample the CLCD dataset from 2001 to 2020 into a resolution of 1 km and then we extracted the cropland data. Before analyzing the impact factors, we first tested these indicators for multicollinearity, indicators with VIF > 10 were excluded to retain significant explanatory variables. Following the principle of data availability and combining with the actual characteristics of the region, 24 key factors including natural and socio-economic factors were finally selected (Table 1). All the data were first reprojected to WGS 1984 UTM Zone 49 N through the ArcGIS 10.8, and then the spatial resolution was resampled to 1 km to maintain data consistency. Moreover, we calculated the average value of the impact factor for each city in the YREB in ArcGIS 10.8 to prevent missing values.

**Table 1:** The bandwidth of each variable in the study.

Variable	Bandwidth
Intercept	124
Zsand	125
Zlai	92
Zmap	126
Zwin	126
Zsrad	126
Zpet	126
Zmrvbf	124
ZPh	126
Zrhu	126
ZSoilMoisture	126
Zsoilerosion	126
Zsoiltype	44
Zbd	126

## 3 Results

### 3.1 Spatial–temporal variability of crop-ESV in YREB

**Figure 2:**
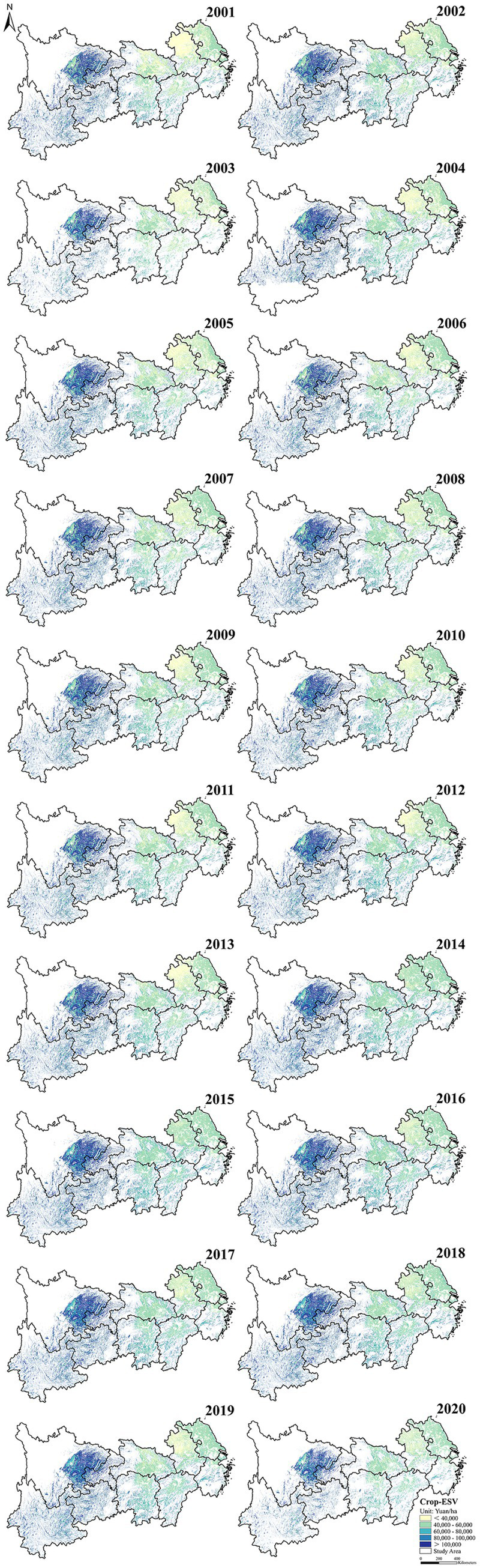
Spatio-temporal variability of crop-ESV in YREB of 2001–2020.

The spatial pattern of Crop-ESV in the YREB between 2001 and 2020 is displayed in Figure 2. In general, the Crop-ESV in the YREB demonstrated obvious spatial heterogeneity. High-value areas were mainly focused on the west of the YREB, especially in Sichuan Province, where the Crop-ESV in some areas exceeds 100,000 yuan/ha. Low-value areas were mainly centered on the east of the YREB, especially in Anhui Province. Notably, the scope of the low-value zone was decreasing over time. Regarding to time, between 2001 and 2010, the Crop-ESV showed a stable or small increase in most regions. While from 2011 to 2020, the Crop-ESV increased significantly in the YREB, especially in Anhui Province. The Sichuan Basin in Sichuan Province has sufficient water and heat conditions, and the cropland is concentrated and continuous (59, 60). The average annual temperature and precipitation range from 14.9 to 18.6°C and 700 to 1,700 mm, respectively (61). The low precipitation zones within the YREB include Anhui and Jiangsu Provinces, with an annual precipitation of about 1,168 mm (62). Additionally, the Sichuan Basin is abundant in purple soil, which is endowed with mineral nutrients for instance phosphorus and potassium, with medium texture, and with excellent water permeability and aeration (63, 64). Gu et al. found that SOC densities in the western part of the YREB (including Sichuan Province) were mostly above 20,000 g C/m^2^, which were higher than those in the northern plains (including Jiangsu, Anhui, and Hubei Provinces) (65). Compared with Sichuan Province, Zhejiang Province and Jiangsu Province are highly urbanized, and cropland has been squeezed by construction land, showing the double pressure of area reduction and quality degradation, weakening the Crop-ESV (66, 67). The annual decreasing rate of cropland in Zhejiang Province, Anhui Province, and Shanghai City reaches 0.46%, and the cropland loss brought by non-agricultural construction tops the list of the YREB. Hence, the pressure on cropland in these areas are increasing sharply (68). Since 2011, Anhui Province has continuously implemented the high-standard cropland construction (69), adjusting and optimizing the cropland ecological pattern, improving the cropland production conditions, and upgrading the cropland quality through multiple ways, like the construction of irrigation and drainage projects, and the prevention and treatment of soil improvement and acidification.^1^^,^^2^

### 3.2 Interannual variation of crop-ESV in YREB

**Figure 3:**
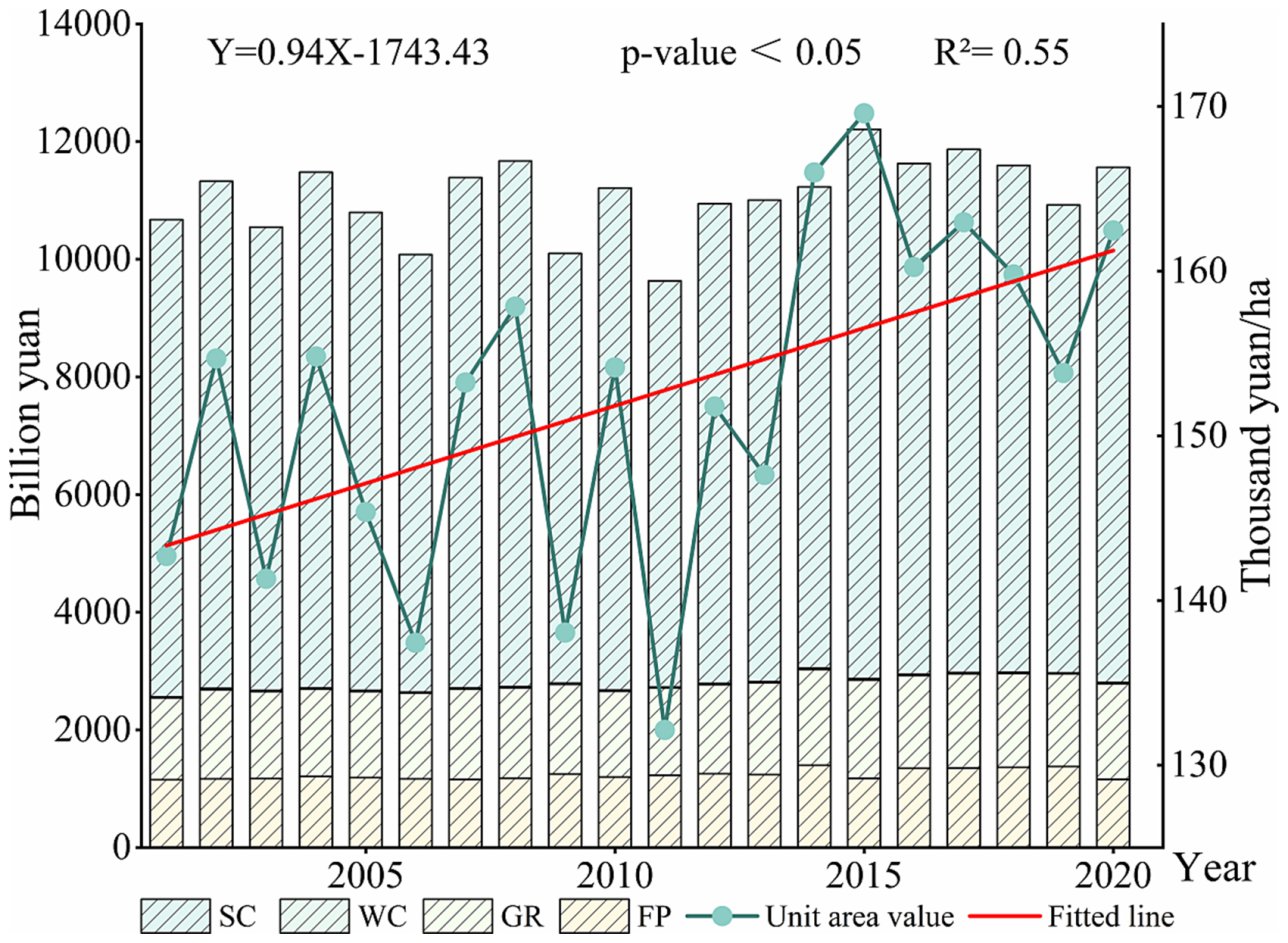
Interannual variation of Crop-ESV from 2001 to 2020. FP, food production; WC, water conservation; GR, gas regulation; SC, soil conservation.

Figure 3 illustrates the interannual changes in the Crop-ESV in the YREB. The overall Crop-ESV of the YREB showed a fluctuating upward trend from 2001 to 2020. It rose from 10,674 billion yuan in 2001 to 11,564 billion yuan in 2020. The highest value occurred in 2015 at 12,211 billion yuan. The lowest value of Crop-ESV occurred in 2011 at 9,633 billion yuan. Generally, the value per unit area also showed a significant up-ward trend, with an increasing trend of 940 yuan/ha per year (*p* < 0.05). Moreover, soil conservation contributed the most to the Crop-ESV in the YREB, followed by gas regulation, then food production, water conservation contributed the least to the Crop-ESV of the YREB. Compared the previous years, the YREB was severely affected by a variety of natural disasters in 2011, mainly including freezing temperatures, snowstorms, floods, droughts and mudslides. According to national natural disaster statistics, Sichuan Province, Hunan Province, Yunnan Province, Guizhou Province and Hubei Province suffered the most disasters in 2011.^3^ Li et al. found that frequent regional and periodic meteorological disasters always happened in 2011 (70). For example, from January to May 2011, the average precipitation in the middle and lower reaches of the Yangtze River was 57% less than that of the same period in many years, and the cropland area in Hunan, Hubei, Jiangxi, Anhui and Jiangsu Provinces suffered from drought amounted to 3,796.97 thousand hectares.^4^ In June 2011, four consecutive floods occurred in the middle and lower reaches of the Yangtze River, with the maximum rainfall amounting to 958 mm, and 280 counties (municipalities) in Anhui, Jiangxi, Hubei, Hunan and Jiangsu Provinces suffered from floods, with 1,929 thousand hectares of crops affected.^5^ The heavy precipitation process led to severe flash floods and mudslides in localized areas. In September 2011, 533 thousand hectares of crops were affected in Sichuan, Chongqing, Hubei, Shaanxi, Henan and other provinces.^6^ The damage to cropland not only directly led to a reduction in food production on cropland in the YREB in 2011, but also indirectly affected the water conservation and soil conservation capacity of cropland in the YREB. All of these may have contributed to a sharp decrease of Crop-ESV in 2011 (46).

**Figure 4:**
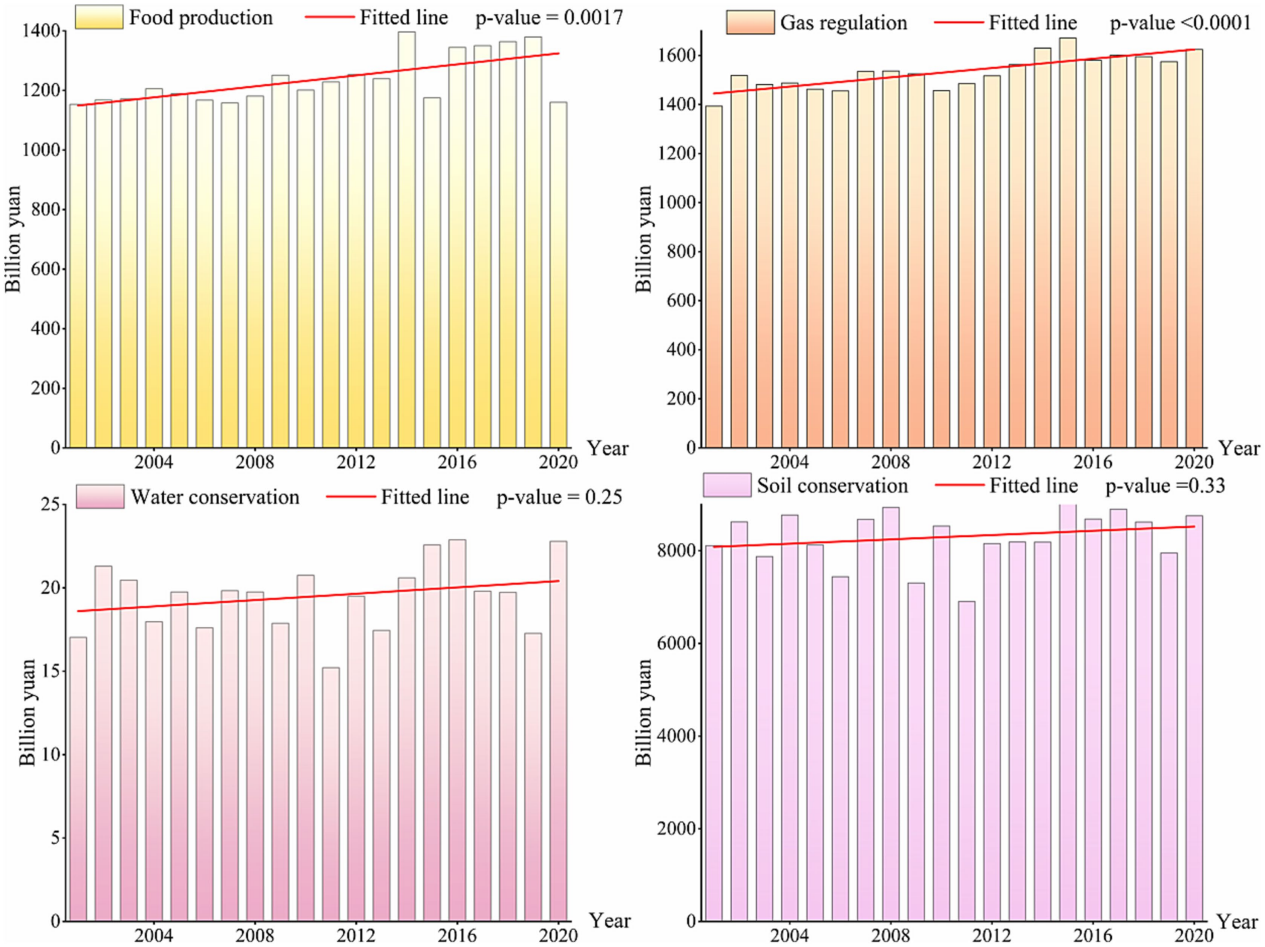
Interannual change of different Crop-ESV.

From 2001 to 2020, the Crop-ESVs of the YREB showed increasing trends (Figure 4). Generally speaking, the values of FP and GR showed significant increasing trends, with FP increasing from 1,153 billion yuan in 2001 to 1,160 billion yuan in 2020 (*p*-value<0.01), GR increasing from 1,393 billion yuan in 2001 to 1,624 billion yuan in 2020 (*p*-value<0.001). Although the values of WC and SC showed increasing trends, the fitted lines for WC (*p*-value = 0.25) and SC (*p*-value = 0.33) were relatively insignificant.

### 3.3 Hot spot of crop-ESV

**Figure 5:**
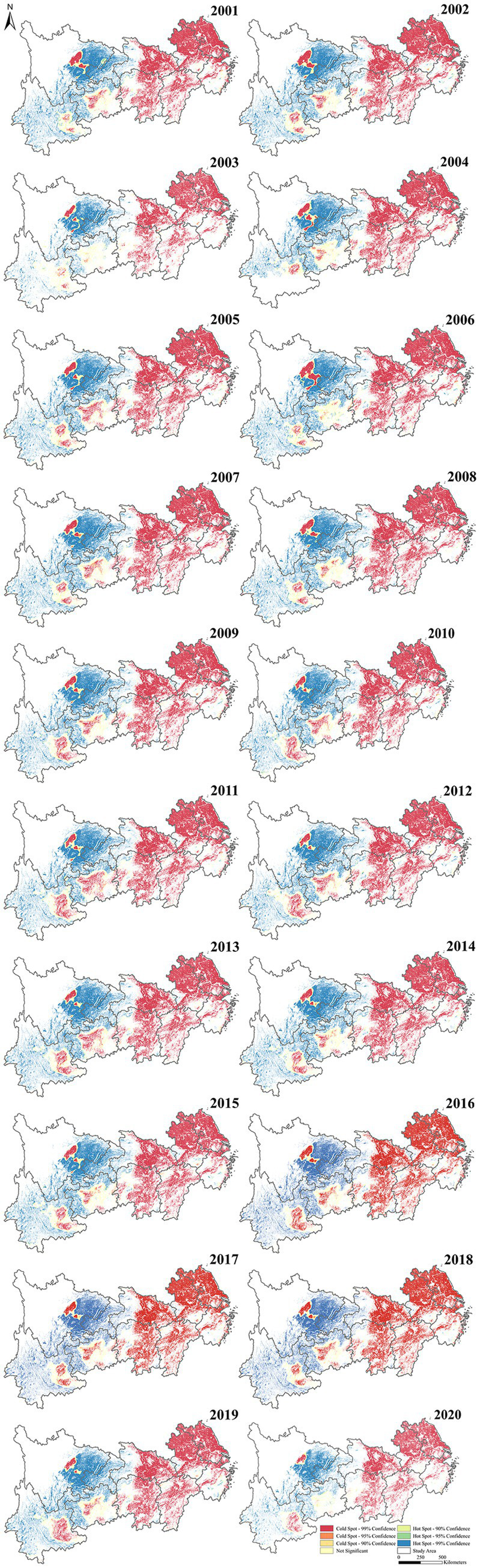
Hot and cold spots of CP-ESV from 2001 to 2020.

The hot spots of the Crop-ESV in the YREB are illustrated in Figure 5. The cold spot zones (red) were mainly clustered in the east and some central zones, while the hot spot zones (blue) were largely localized in the west zones. The distribution of cold spot regions was relatively stable, primarily concentrated in Jiangsu, Anhui, Zhejiang and Jiangxi provinces. The hot spot regions were mostly distributed in Sichuan and Yunnan provinces. Besides that, the cold spot regions of Crop-ESV had been shrinking during the past 20 years.

## 4 Discussion

### 4.1 Spatial–temporal trend of crop-ESV in YREB

**Figure 6:**
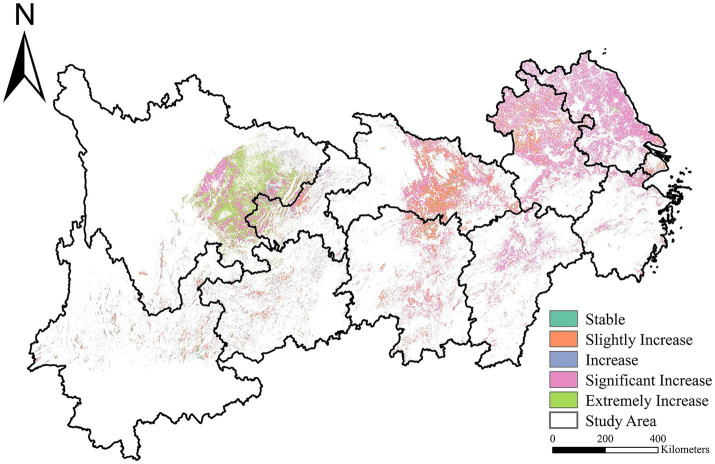
Spatially explicit trend of Crop-ESV.

Using the Sen-MK trend assessment, we analyzed the temporal tendency of the Crop-ESV in the YREB. Generally, the Crop-ESV in the YREB showed a trend of high in the west and low in the east and demonstrated obvious spatial heterogeneity (Figure 6). Furthermore, the Crop-ESV from 2001 to 2020 displayed an overall rising trend in the YREB, with a slight increase in Crop-ESV in Hubei Province. Jiangsu, Anhui, Zhejiang, Jiangxi, Hunan and Chongqing provinces increased significantly. In addition, the Crop-ESV increased sharply in Yunnan Province, especially in Sichuan Province. This result is consistent with the reported (8, 71, 72).

Since the 20th century, China has continuously deployed lots of measures to strengthen the quality of cropland and optimize its ecosystem functions. In 2004, the No. 1 Document issued by the Chinese government clearly stated that “the quality of cropland should be continuously improved” and “governments at all levels should earnestly implement the strictest system of protecting cropland”.^7^ In 2008, China government proposed “adhering to the strictest system of cropland protection, and resolutely guarding 1.8 billion mu of cropland red line” (see Footnote 6). In 2012, the construction of high-standard farmland in China began to be promoted.^8^ In 2019, the Ministry of Agriculture and Rural Development issued an implementation opinion on supporting the agriculture and rural areas green development in the YREB. It stated that the strictest system of cropland protection should be adhered to, and that the policy measures for the special protection of permanent basic cropland should be comprehensively implemented.^9^ In 2024, the YREB-Yangtze River Basin Land Spatial Planning (2021–2035) proposes that by 2035, the cropland retention in the Yangtze River Economic Belt-Yangtze River Basin will be no less than 39.98 million hectares.^10^ Provinces and cities within the YREB have actively responded to the call to continuously implement cropland protection policies and improve cropland protection measures, resulting in an increase in the Crop-ESV within the YREB region.

### 4.2 The impact pathways of different factors on spatial–temporal variability of crop-ESV

**Figure 7:**
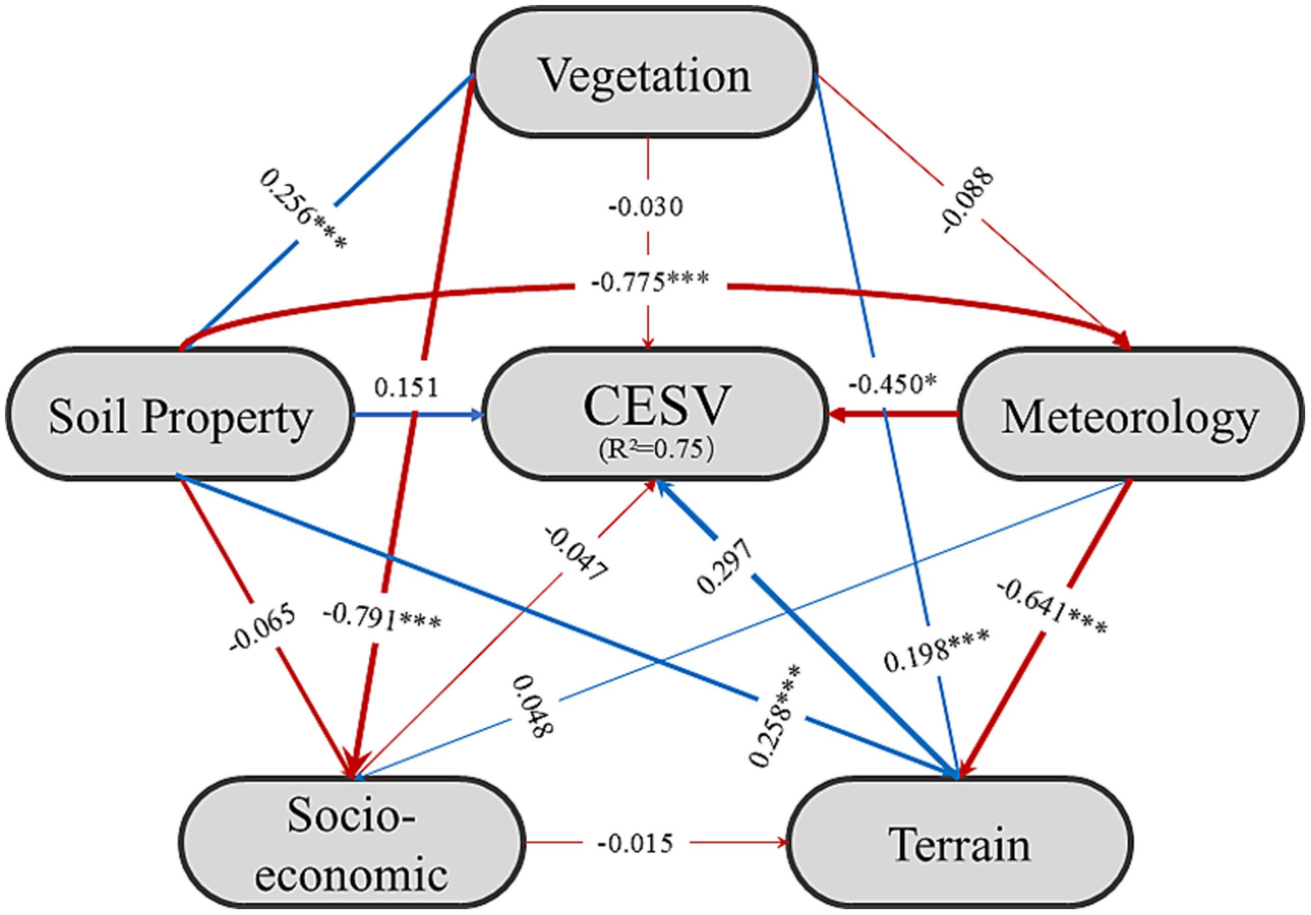
Crop-ESV pathways and their drivers identified by SEM. Numbers in lines denote path efficiencies. Blue lines denote positive path coefficients or loadings and red lines denote negative path coefficients or loadings. The width of each line is proportionate to the respective path coefficient or load. Note: *** denotes *p* < 0.0001; ** denotes *p* < 0.01; * denotes *p* < 0.05.

Changes in Crop-ESV in the YREB were affected by a synthesis of natural and socio-economic drivers (Figure 7). Using SEM, our study investigated the implications of diverse factors on Crop-ESV in the YREB. The explained variance of the constructed SEM model for Crop-ESV is 0.75, indicating that the model has a strong explanation of Crop-ESV. Terrain (0.297) and soil property (0.151) are the main positive drivers of Crop-ESV. Terrain factors such as DEM and slope have a dual effect on Crop-ESV. Proper slope can regulate surface runoff and retain soil, thus increasing WC and SC (73). Whereas, as slope increases, surface runoff speeds up, water reserve capacity decreases, and soil erosion increases significantly, leading to a decrease in FP, WC and SC (74, 75). The Sichuan basin in the west of the YREB is gently sloping and flat, with superior soil conditions. Despite the downstream areas have flat terrain, they have experienced severe cropland fragmentation due to rapid urbanization. Additionally, the middle and lower reaches of the YREB have faced soil heavy metal pollution and serious soil degradation (76). Soil property like soil organic carbon, it is not only an important element of the global carbon stock, but also has a significant impact on soil fertility and quality (77, 78). Higher soil organic carbon content in Sichuan Province than other region in the YREB not only has a direct effect on GR, but also indirectly increases FP and WC (79, 80). While meteorological conditions (−0.450) and vegetation (−0.030) might reduce Crop-ESV. This result was also found in the ecosystem service values of the HuangHuaihai Plain and the Pearl River Delta. Qiao et al. revealed that precipitation and NDVI were the main negative factors influencing the intensity of ecosystem service relationship in the HuangHuaihai Plain (81, 133). Zou et al. found that NDVI mainly played a negative driving role in the Pearl River Delta, and its inhibitory effect generally showed a weakening trend (82). Meteorological factors include mean annual temperature, mean annual pressure. Moderate temperature extends the crop growing period (83), promotes the soil organic matter decomposition (83), thereby increasing FP. If the temperature is too cold or too hot, it may affect crop growth and reduce FP (84–86). Evidence had shown that the extreme temperature had led the reduced production in Jiangsu Province, Hunan Province, and Jiangxi Province.^11^^,^^12^ Guo et al. revealed that extreme high temperature and extreme low temperature both had a significant negative effect on food production (87). Additionally, it may accelerate the soil moisture evaporation, which may lead to soil drought or increased salinization, resulting in soil degradation, finally influencing SC (88). Chen et al. found that climate warming will escalate soil erosion and damage soil quality (89). Mean annual pressure can also negatively affect the Crop-ESV. Persistent low pressure is often accompanied by heavy precipitation, which can lead to cropland flooding (90). In 2012, the heavy precipitation made the cropland flooding in Jiangxi Province and Hunan Province, reducing the crop production.^13^ Feng et al. (2025) disclosed that high temperature, high solar radiation, heavy rainfall, and strong wind may lead to soil degradation (91). Persistent high pressure can lead to drought, which can also reduce FP (92). According to the China Meteorological Administration, high pressure is one of the main climate systems in summer, and high pressure in China always leads to high temperature and agriculture drought. Under the influence of high pressure, southern and northern agricultural areas are prone to moderate or even severe agricultural drought (93), which reduces crop yields.^14^ Vegetation factors include the enhanced vegetation index (EVI) and the normalized difference vegetation index (NDVI). When the NDVI and EVI values are low, they indicate poor vegetation growth on cropland, which may lead to lower FP (34, 94). At the same time, reduced vegetation cover can make soil more susceptible to erosion, leading to reduced soil fertility and affecting SC (95). Socio-economic activities also have a negative impact on Crop-ESV (−0.047), indicating that there is a certain contradiction between economic development and ecological protection (96, 97). The over-exploitation of mineral resource (phosphorus mining) in Yunnan, Sichuan, and Guizhou Provinces has led to soil heavy pollution, resulting in a decline in the Crop-ESV (98). In the middle reaches of the YREB, Hubei, Hunan and Jiangxi Provinces have encroached on high-quality cropland with economic development, leading to a decrease in cropland area (99). In addition, the excessive use of chemical fertilizers and pesticides has led to serious surface source pollution in these provinces (100). As a result, the Crop-ESV declined. The industrial development of the lower reaches of the YREB has led to a large reduction of cropland area (101). At the same time, industrial pollution and urban sewage irrigation have led to the accumulation of heavy metals and organic pollutants on cropland, affecting the Crop-ESV in the YREB (78).

### 4.3 Spatially-varying dominators of spatial–temporal variability of crop-ESV

In our research, we employed the MGWR model to gain a deeper understanding of the spatially-varying influence of each driver in Crop-ESV. According to the results in Table 2, our constructed MGWR model has the strongest explanatory power compared with OLS and GWR. The AICc value is 166.52, much lower than the OLS (181.16) and the GWR (203.59). Furthermore, the adjusted R^2^ of MGWR is 0.84, 9% higher than OLS, 3% higher than GWR. At the same time, the Residual Sum of Squares (RSS) of MGWR is 16.80, which indicates that the model has high explanatory accuracy (Table 3).

**Table 2:** Data source in the research.

Driving factors		Abbreviation	Sources
Meteorological data	Mean annul precipitation	MAP	http://www.geodata.cn
	Mean annul temperature	MAT	http://www.geodata.cn
	Mean annul pressure	PRE	https://www.resdc.cn
	Mean annul wind	WIN	https://www.resdc.cn
	Mean annul relative humidity	RHU	https://www.resdc.cn
	Potential evapotranspiration	PET	http://www.geodata.cn
	Solar radiation	SRAD	https://data.tpdc.ac.cn
Land cover data	Map of cropland		Yang and Huang (58)
Soil data	Soil organic carbon	SOC	https://doi.org/10.3334/ORNLDAAC/1247
	Soil texture		https://www.resdc.cn
	Soil Type	ST	https://www.resdc.cn
	Soil Erosion	SE	https://www.resdc.cn
	Soil Moisture	SM	https://doi.org/10.24381/cds.e2161bac
	Soil bulk density	BD	https://doi.org/10.3334/ORNLDAAC/1247
	Soil pH	pH	http://poles.tpdc.ac.cn
	Soil saturated hydraulic conductivity	Ksat	https://www.isric.org
Vegetation data	Leaf Area Index	LAI	https://doi.org/10.1029/2012JG002084
	Normalized Difference Vegetation Index	NDVI	https://www.resdc.cn
	Enhanced Vegetation Index	EVI	https://www.resdc.cn
Terrain data	Digital elevation model	DEM	https://www.gscloud.cn
	Slope	Slope	This Study
	Multi-resolution valley bottom flatness	MRVBF	This Study
Socio-economic data	Population density	PD	https://www.worldpop.org
	Gross Domestic Product	GDP	https://www.resdc.cn
	Nighttime lighting	NTL	https://www.resdc.cn

**Table 3:** Performance of different models.

Model	OLS	GWR	MGWR
R^2^	0.78	0.85	0.87
R^2^ Adjusted	0.75	0.81	0.84
AICc	203.59	181.16	166.52
RSS	28.19	19.82	16.80
ENP	14.00	21.97	24.04

**Figure 8:**
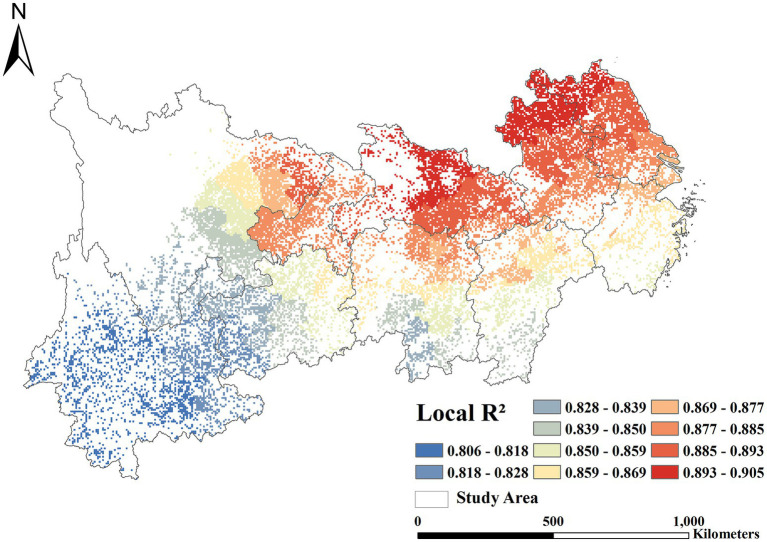
The local R^2^ of MGWR.

In addition, based on local R^2^ map (Figure 8), MGWR is able to accurately and reliably reflect the spatial heterogeneity of drivers and provide clearer spatial details. As presented in Figure 8, the R^2^ in the YREB shows a gradient from high in the northeast to low in the southwest. The high-fitting areas are largely concentrated in Jiangsu, northern Anhui and central Hubei, which have been highly urbanized in recent years, and the Crop-ESV is less affected by natural fluctuations (102). In addition, these areas have a high agricultural intensification level, a relatively single land use type, and a land use management practices convergence (103). Therefore, the fitting result is higher than that of other regions in the YREB. The medium fitting zone is distributed along the Yangtze River, covering parts of Hunan, Jiangxi and Zhejiang, and the topography of this region transitions from plain to hilly. Furthermore, they are in the city-nature ecological transition zone, with both agricultural activities and natural disturbances, such as seasonal flooding (104). The low-fitting areas are scattered in the Yunnan-Guizhou Plateau. Affected by the complex topography and karst landscape of the Yunnan-Guizhou Plateau, the Crop-ESV was lowly fitted to the influencing factors (105).

**Figure 9:**
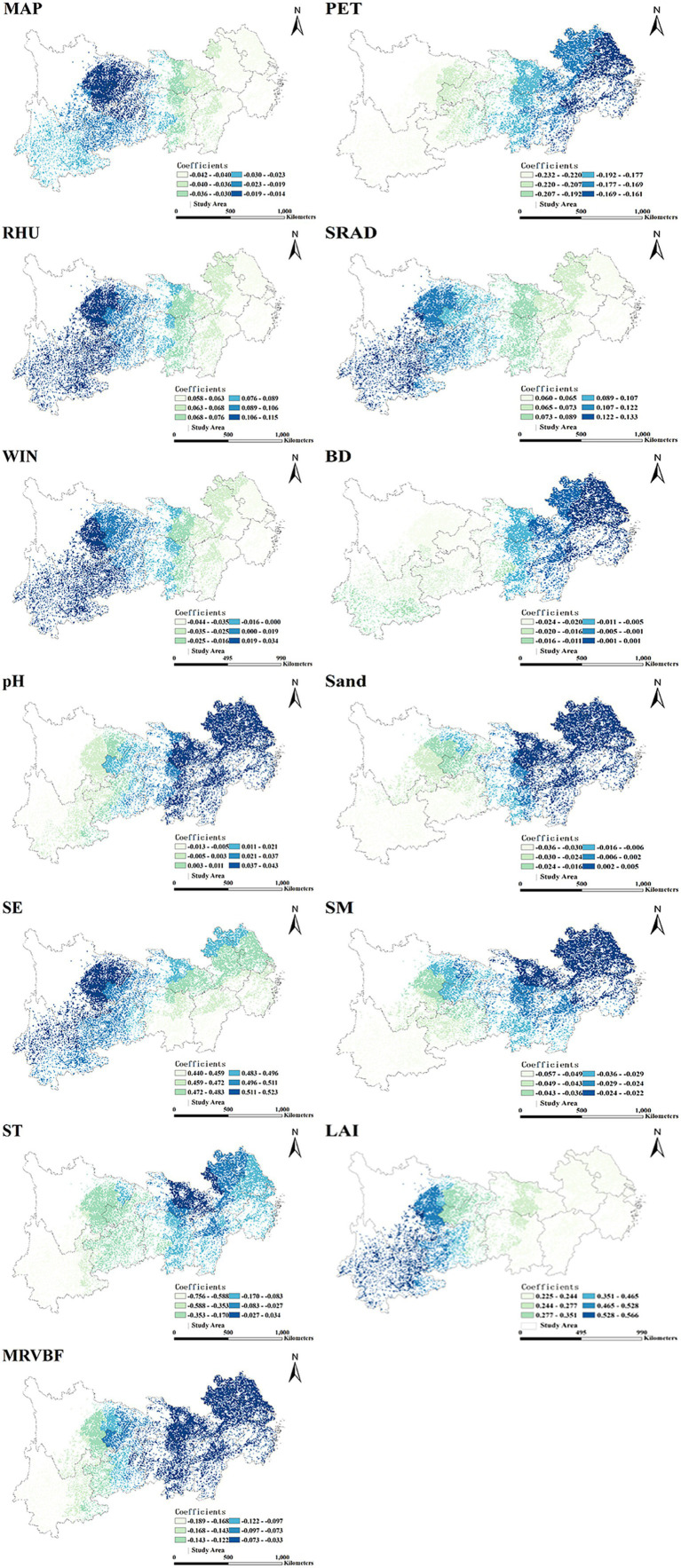
Spatial distribution of coefficients of the driving factors estimated by the MGWR model. Abbreviation: MAP, mean annual precipitation; PET, potential evapotranspiration; RHU, mean annul relative humidity; SRAD, solar radiation; WIN, Mean annul wind; BD, soil bulk density; pH, soil pH; SE, soil erosion; SM, soil moisture; ST, soil type; LAI, leaf area index; MRVBF, multi-resolution valley bottom flatness.

Specifically, our constructed MGWR model accurately revealed the geographic gradient characteristics of the factors on the spatial scale (Figure 9). Soil bulk density (BD), Mean annual precipitation (MAP), MRVBF, and potential evapotranspiration (PET) showed significant negative effects on Crop-ESV, while the effects of soil pH and Sand were obviously regionally heterogeneous, with the low-value area centered in medial Yangtze River reaches and the high-value zone distributed in the Yunnan-Guizhou Plateau region. In contrast, LAI, RHU and solar radiation (SRAD) were strongly positively correlated with Crop-ESV.

Among them, the meteorological factors essentially influence Crop-ESV in the YREB. The YREB has summers with more precipitation and higher temperature, mild winters with little rain (106). RHU and SRAD are positively correlated with Crop-ESV in this study, suitable RHU and SRAD favor biological reproduction, promote soil nutrient cycling, and maintain ecological stability and diversity (107, 108). While MAP and PET were negatively correlated to Crop-ESV, previous study had demonstrated that the multifunctionality of ecosystems tends to diminish under high precipitation intensity (34). In addition, enhanced potential evapotranspiration increases ecosystem water expenditure, which is prone to resource scarcity and vegetation degradation, and thus negatively affects the Crop-ESV (109).

Soil properties also play a significant role in maintaining and enhancing Crop-ESV. There is a negative correlation between soil moisture (SM) and Crop-ESV as shown in Figure 9. The soil erosion (SE) of the YREB is developed in a “belt” shape, showing the state of high east and low west. Improper soil moisture will adversely affect the vegetation growth and soil health, leading to the reduction of Crop-ESV (110). BD, sandy soil and soil type (ST) all showed more negative effects on Crop-ESV in the western region compared to the eastern region. The high value area of Crop-ESV in the western region was dominated by purple soils with neutral soil pH, higher BD and lower sand content (111). In contrast, the low value area of Crop-ESV in the eastern region is dominated by red soils with acidic soil pH, moderate soil BD, and high sand content (77). Purple soils in the western region are more sensitive to changes in BD, sandy soils, and soil pH than those in the eastern region (112). Therefore, these factors have a greater negative impact on the western region.

The positive contribution of vegetation factors to Crop-ESV and their values has been much mentioned in previous studies (113). The Crop-ESV can be significantly affected by vegetation cover due to the differences in cover of different vegetation types (8). In this study, Yunnan, Sichuan and Guizhou, due to high vegetation cover, provide more Crop-ESV. While Zhejiang, Shanghai and Jiangsu are economically developed and rapidly urbanized, and human activities interfere more with natural ecosystems, resulting in lower vegetation cover and reduced the Crop-ESV (114).

In this study the terrain factor MRVBF dominated the spatial variation of Crop-ESV, and the influence of terrain factors on Crop-ESV was closely related to vegetation types and anthropogenic factors. MRVBF was negatively correlated with Crop-ESV, especially in the eastern coastal region, where the flat topography leaded to stronger sedimentation and higher MRVBF values. Thus, the negative effect of MRVBF on Crop-ESV was greater in the east area. While in the west area, where the complex topography and lower development intensity leaded to lower MRVBF values. Consequently, the negative effect of MRVBF on Crop-ESV was relatively small (115).

## 5 Implications of current study

The YREB spans the east, the middle and the west regions in China, and has distinctive advantages and great development potential. In order to enhance the Crop-ESV in the YREB and to enhance the sustainable agricultural advancement of the YREB. We make the following recommendations.

Firstly, the Crop-ESV in the YREB had been increasing over the past two decades, which indicated the effectiveness of the policies on cropland protection and ecological conservation in this area. At the same time, although the values of FP, GR, WC and SC had all increased, the trend of increase in WC and SC was not significant compared with that of FP and GR. This reminds that while maintaining the values of FP and GR, we should focus on increasing the values of WC and SC.

Secondly, according to the results of the hotspot analysis, the Crop-ESV within the YREB showed obvious spatial heterogeneity. Therefore, more responsive and effective measures for the protection of cropland and zoning plans for agricultural production should be developed (8). Even though provinces and cities within the YREB have been persistently pursuing cropland protection policies and the area of cold spot regions was decreasing, continuous attention should also be maintained on cold spot regions. For areas along the eastern coast, attention should be paid to the balance between ecological protection and economic development, and urban agriculture-ecological protection zones can be designated to avoid a decline in the Crop-ESV because of over-exploitation (116–118). For the cropland fragmentation in the middle and lower reaches of the YREB, the implementation of cropland consolidation plans, cropping structures optimization or cropland replacement can help reduce cropland fragmentation and improve land use efficiency (119, 120). Additionally, industrial production activities are the main factor affecting the accumulation of soil heavy metals in the eastern provinces, governments should formulate and strictly enforce policies for the prevention and control of soil heavy metal pollution (76). For central areas, for instance, Jiangxi, Hunan and Hubei provinces, which have been affected by soil acidification and soil degradation problems (121–123). In these areas, organic fertilizers, straw, green manure can be applied to increase the soil organic matter content, enhance the soil buffering capacity, and reduce soil acidification rate (77, 124). Zhao et al. found that the application of organic fertilizers significantly increased the yields of rice, wheat and maize by 47.0, 78.0 and 76.9%, respectively. In addition, straw fertilization not only significantly increased the contents of SOC, but also markedly increased the yields of rice, wheat and maize by 24.5, 10.1 and 12.1%, respectively (125). On this basis, it is also possible to track and monitor the effects of acidified cropland management in order to scientifically assess the effectiveness of implementation. For areas with high Crop-ESV, they can be set as protection areas. In addition, for some areas where the Crop-ESV is extremely low, the policy of returning cropland to forests and grasses can also be implemented in order to realize sustainable development.

Thirdly, when reclaiming cropland, consideration should be given to the natural geographical conditions of each area. Crops should be planted in areas with gentle slopes, favorable climate and rich soil organic carbon content. When the slope is greater than 25°, we should implement the policy of returning cropland to forest.^15^ Since 2000, 153,694 km^2^ of cropland has been returned to forests in the YREB (126). Yin et al. found that the policy of returning cropland to forests has helped to maintain a good level of ecological security. Although the policy has led to a reduction of cropland area and a decrease in Crop-ESV, an increase in forest facilitates the provision of ecosystem services such as carbon storage and habitat quality (127). We should also emphasize the protection of crops and provide them with a good growing environment in order to increase food production.

Our study mapped the spatio-temporal distribution of Crop-ESV by integrating Sen-MK, SEM, and MGWR at a resolution of 1KM, and deeply explained the driving mechanisms of Crop-ESV. It not only proved the increasing trend of Crop-ESV in key regions, but also deepened the understanding of multifunctional cropland and the complexity of the driving mechanism, and can also provided corresponding experience and solutions for similar regions around the world.

## 6 Limitations and future research progress

Although this study has made some progress, like other research, it still has several limitations. In future studies, we need to consider the following factors comprehensively. Firstly, as the core dataset, inherent differences in the spatial and spectral resolution of the original remote sensing images may introduce significant biases in subsequent analyses (128, 129). Secondly, in the model analysis, we did not account for the effects of local factors on Crop-ESV (such as the impact of topographical differences between eastern and western regions on Crop-ESV). Thirdly, constrained by data availability, our assessment of Crop-ESV failed to encompass certain services related to soil pollution (130), water eutrophication (131), and biodiversity (132), which may lead to an overestimation of the final ESV estimates. Finally, when analyzing the factors affecting the Crop-ESV, we did not take policy factors (cropland protection policies) into account.

## 7 Conclusion

Grounded in multi-sources data, our study mapped the spatio-temporal distribution of Crop-ESV in the YREB in the past two decades. And then, we revealed the implications of diverse influencing factors on the Crop-ESV. Our findings indicated that the total Crop-ESV in the YREB increased over the past two decades, especially the value of FP and GR. Meanwhile, the Crop-ESV in the YREB showed obvious spatial heterogeneity, with a trend of high in the west and low in the east. The hotspot of Crop-ESV in the YREB expanded from 2001 to 2020. The distribution of hotspot and cold spot areas tended to be consistent with the distribution of Crop-ESV in the YREB. Among the influencing factors affecting the Crop-ESV in the YREB, meteorological factors, vegetation factors, and socio-economic factors had a negative influence on Crop-ESV. Soil property factors had a positive effect on Crop-ESV. Terrain factors such as DEM and slope have a dual effect on Crop-ESV. Proper slope and gentle terrain have a contributing effect on the Crop-ESV; however, steep slope or terrain have a negative effect on the Crop-ESV. Our results can not only establish a scientific grounding for the food production increasement, agriculture sustainable development, and maintain the food security, which corresponding to the zero hunger of the SDGs, but also enhance the bioecological functions in the YREB and similar regions around the world.

## Data Availability

The original contributions presented in the study are included in the article/Supplementary material, further inquiries can be directed to the corresponding author.

## Footnotes

^1^ https://slt.ah.gov.cn/public/21731/121432951.html

^2^ https://nync.ah.gov.cn/public/7021/56308051.html

^3^ http://www.caderm.org/site/content/329.html

^4^ https://www.gov.cn/jrzg/2011-09/13/content_1946350.html

^5^ http://mwr.gov.cn/zzsc/tjgb/zgshzhgb/2011/files/basic-html/page22.html

^6^ https://www.cma.gov.cn/2011xwzx/2011xqxxw/2011xqxyw/201201/t20120110_158700.html

^7^ https://www.gov.cn/gzdt/2009-09/30/content_1430754.html

^8^ https://zrzy.hebei.gov.cn/heb/gongk/gkml/kjxx/kjfz/10971151685173919744.html

^9^ https://www.gov.cn/gongbao/content/2019/content_5361805.html

^10^ https://f.mnr.gov.cn/202402/t20240211_2837651.html

^11^ https://www.cma.gov.cn/2011xzt/20120816/2012081601_2_1_1/201208160102/201210/t20121031_188800.html

^12^ https://www.cas.cn/kj/201708/t20170817_4611466.shtml

^13^ https://www.cma.gov.cn/2011xzt/2012zhuant/20120531_1/2012053103/202111/t20211104_4204311.html

^14^ https://www.cma.gov.cn/ztbd/2023zt/20230324/2023032406/202309/t20230908_5764002.html

^15^ https://www.ndrc.gov.cn/hdjl/yjzq/201604/W020210709577443067272.pdf
