# Supplementary material for: Spatio-temporal dynamics and drivers of cropland ecosystem services value during the past two decades in Yangtze river economic belt, China
Source: Front Public Health. 2025 Jun 27;13:1622093. doi: 10.3389/fpubh.2025.1622093 (PMC12245870; doi:10.3389/fpubh.2025.1622093)
Supplement: Supplementary file 1 [file Table_1.docx]

Supplementary Material

# Detailed evaluation methods of cropland ecosystem services value

## Food Production (FP)

Food production is the most fundamental and essential cropland ecosystem service. The Yangtze River Economic Belt (YREB) is one of the most important grain-producing regions in China. Consequently, we have to assess the food production in the YREB. The annual province food production was allocated to the cropland grids with the following formula (1):

$$\begin{aligned} F_{i}=\frac{{NDVI}_{i}}{{NDVI}_{mean}}\times f\#\left( 1 \right) \end{aligned}$$

$\text{F}_{\text{i}}$is the annual food yield of grid $i$ (kg/ha), $\text{NDVI}_{\text{i}}$ refers to the NDVI value of the grid $i$, $\text{NDVI}_{\text{mean}}$ is the average NDVI of study area, and $\text{f}$ refers to the average yield of study area for each year (kg/ha).

What’s more, this paper utilized the market value approach to calculate the monetary value of annual food production based on the 2020 food sale price. The formula is as below:

$$\begin{aligned} V_{F,i}=F_{i}\times P_{F}\#\left( 2 \right) \end{aligned}$$

$\text{V}_{\text{F,}\text{i}}\text{ }$means the food production value of grid $i$ each year (yuan/ha), $\text{P}_{\text{F}}\text{ }$is the 2020 food price. According to the national cost-benefit compilation of agricultural products, food price was 2.449 yuan/kg in 2020.

## Gas Regulation (GR)

The GR value covered the dual paths of carbon dioxide fixation and oxygen release (2), which were monetized with reference to the Nordic carbon tax standard and the cost of industrial oxygen production, respectively. The physical quantity of CO_2_ fixation and O_2_ release in cropland ecosystem was calculated based on net primary productivity (NPP) and crop physiological processes. It has been shown that 1gC is equivalent to 2.2g organic matter (3).1kg of dry matter can fix 1.62g CO_2_ and release 1.19g O_2_ according to the equation for plant photosynthesis. Thus, the formulas are as follows:

$$\begin{aligned} G_{c,i}={NPP}_{i}\times2.2\times1.62\#\left( 3 \right) \end{aligned}$$

$$\begin{aligned} G_{o,i}={NPP}_{i}\times2.2\times1.19\#\left( 4 \right) \end{aligned}$$

$\text{G}_{\text{c,i}}$refers to the CO_2_ fixation quantity of grid $i$each year(g/m^2^), $\text{G}_{\text{o,i}}$is the O_2_ release quantity of grid $i$each year, $\text{NPP}_{\text{i}}$is the net primary productivity (gC/m^2^).

The NPP is estimated by the Carnegie-Ames-Stanford Approach (CASA). The CASA model is widely used at regional scales due to its ability to calculate parameters easily and to reveal the temporal dynamics of NPP (4,5), which consists of two basic formulas:

$$\begin{aligned} \text{NPP}_{\text{i}}\text{=}\text{APAR}_{\text{i}}\text{×}\text{ε}_{\text{i}}\#\left( 5 \right) \end{aligned}$$

$$\begin{aligned} \text{APAR}_{\text{i}}\text{=}\text{SOL}_{\text{i}}\text{×}\text{FPAR}_{\text{i}}\text{×0.5}\#\left( 6 \right) \end{aligned}$$

$\text{APAR}_{\text{i}}$is the absorbed photosynthetically active radiation, $\text{ε}_{\text{i}}$ is the actual light use efficiency, $\text{SOL}_{\text{i}}$represents the solar radiation, $\text{FPAR}_{\text{i}}$represents photosynthetically active radiation absorption coefficient.

This paper adopted the carbon tax and industrial cost of oxygen production to calculate the value of CO_2_ fixation and O_2_ release, respectively. The formulas are as follows:

$$\begin{aligned} V_{c,i}=G_{c,i}\times P_{c}\#\left( 7 \right) \end{aligned}$$

$$\begin{aligned} V_{o,i}=G_{o,i}\times P_{o}\#\left( 8 \right) \end{aligned}$$

$$\begin{aligned} V_{G,i}=V_{c,i}+V_{o,i}\#\left( 9 \right) \end{aligned}$$

$\text{V}_{\text{c,i}}\text{ }$represents the CO_2_ fixation value of grid $\text{i }$each year (yuan/ha), $\text{P}_{\text{c}}$ refers to the cost of oxygen production. $\text{V}_{\text{o,i}}$ is the O_2_ release value of grid $\text{i }$each year (yuan/ha), $\text{P}_{\text{o}}$refers to the Sweden carbon tax, $\text{V}_{\text{G,i}}$is the annual gas regulation value. The Sweden carbon tax is derived from the World Bank (https://www.worldbank.org/). Oxygen production cost refers to (3).

## Water Conservation (WC)

Cropland ecosystems are able to intercept and infiltrate precipitation through crops and soils, thereby reducing runoff generation and surface water loss. Water conservation is key to filtering out pollutants, maintaining clean drinking water and ecosystem health.Not only does it regulate runoff and store floodwaters, but it also provides adequate water resources both within and outside the crop ecosystem. The WC value was estimated by integrating the water balance model and the shadow engineering method, and the ecological benefits are discounted by the construction cost per unit of reservoir capacity.

The water balance model was used to calculate water conservation in cropland ecosystem. Specifically, the water yield of cropland ecosystem is first calculated using the InVEST model, and then adjusted according to topography, soil infiltration and surface runoff coefficients (6). The formulas are as follows:

$$\begin{aligned} Y_{i}=\left( 1-\frac{{AET}_{i}}{P_{i}} \right)\times P_{i}\#\left( 10 \right) \end{aligned}$$

$$\begin{aligned} \frac{{AET}_{i}}{P_{i}}=1+\frac{{PET}_{i}}{P_{i}}-\left[ 1+\frac{\left( {AET}_{i} \right)^{\omega}}{P_{i}} \right]^{1/\omega}\#\left( 11 \right) \end{aligned}$$

$\text{Y}_{\text{i}}$represents the water yield of grid $\text{i}$ each year (mm), $\text{P}_{\text{i}}$means the precipitation of grid $\text{i}$ (mm), $\text{AET}_{\text{i}}$is the actual evapotranspiration of grid $\text{i}$ (mm), $\text{PET}_{\text{i}}$ is the potential evapotranspiration of grid $\text{i}$ (mm), $\text{ω}$ is a non-physical parameter that describes the natural environmental properties.

$\begin{aligned} W_{i}=min\left( 1,\frac{249}{V} \right)\times min\left( 1,\frac{0.9TI}{3} \right)\times min\left( 1,\frac{Ksat}{300} \right)\times Y_{i}\#\left( 12 \right) \end{aligned}$ the annual water conservation of grid $\text{i}$ (mm), $\text{V}$ represents the runoff velocity coefficient, $\text{TI}$ refers to the topographic index, $\text{Ksat}$ is the saturated soil hydraulic conductivity (mm/d).

The water conservation value is computed by the shadow engineering method using the following formula:

$$\begin{aligned} V_{w,i}=W_{i}\times P_{w}\#\left( 13 \right) \end{aligned}$$

$\text{V}_{\text{w,i}}$means the water conservation value of grid $\text{i}$ each year (yuan/ha), $\text{P}_{\text{w}}$is the cost of reservoir construction. The cost of reservoir construction is taken from (4).

## Soil Conservation (SC)

Frequent extreme weather events and increased anthropogenic disturbances lead to an increased risk of soil erosion, which adversely affects cropland ecosystems (7). The implementation of soil conservation measures ensures the sustainability of agricultural production, maintains the health of ecosystems and reduces the risk of flooding. The SC value consisted of sediment reduction and nutrient retention, with the former using the engineering replacement cost method and the latter combining the market price of elements and soil remediation costs.

The physical quantity of soil conservation in cropland ecosystem is estimated by the Revised Universal Soil Loss Equation (RUSLE) with the following formula:

$$\begin{aligned} S_{i}=R_{i}\times K_{i}\times{LS}_{i}\times\left( 1-C_{i}\times P_{i} \right)\#\left( 14 \right) \end{aligned}$$

$\text{S}_{\text{i}}$is the soil conservation of grid $\text{i}$ (t ha^-1^ yr^-1^), $\text{R}_{\text{i}}$ is the rainfall erosivity factor (MJ mm ha^-1^ h^-1^ yr^-1^), $\text{K}_{\text{i}}$ represents the soil erodibility factor (t ha h ha^−1^ MJ^−1^ mm^−1^), $\text{LS}_{\text{i}}$ refers to the slope length and steepness factor, $\text{C}_{\text{i}}$is the cover and management factor, $\text{P}_{\text{i}}$ is the support practice factor. All factors were calculated with reference to previous studies (8-13). The formulas are expressed as follows:

$$\begin{aligned} R_{i}=\sum_{j=1}^{12} -1.5527+1.792P_{ij}\#\left( 15 \right) \end{aligned}$$

| $\begin{aligned} K_{i}=\left\{ 0.2+0.3exp\left[ -0.0256\times{SAN}_{i}\left( 1-\frac{{SIL}_{i}}{100} \right) \right] \right\}\times\left( \frac{{SIL}_{i}}{{CLA}_{i}+{SIL}_{i}} \right)\# \end{aligned}\times\left[ 1-\frac{0.25{SOM}_{i}}{{SOM}_{i}+\exp\left( 3.72-2.95{SOM}_{i} \right)} \right]\times\left[ 1-\frac{0.7\times\left( 1-{{SAN}_{i}}/{100} \right)}{\left( 1-{{SAN}_{i}}/{100} \right)+\exp\left( -5.51+22.9\times\left( 1-\frac{{SAN}_{i}}{100} \right) \right)} \right]\times0.1317$ | (16) |
| --- | --- |

$$\begin{aligned} S_{i}=\left\{ \begin{aligned} 10.8\sin\theta+0.03 \theta<5 \\ 16.8\sin\theta-0.5 5\leq\theta\leq10\#\#\# \\ 21.91\sin\theta-0.96 \theta>10 \end{aligned} \right.\#\left( 17 \right) \end{aligned}$$

$$\begin{aligned} L_{i}=\left\{ \frac{\lambda_{i}}{22.13} \right\}^{\left[ {\beta_{i}}/\left( 1+\beta_{i} \right) \right]}\#\left( 18 \right) \end{aligned}$$

$$\begin{aligned} \beta_{i}=\left( {\sin\theta}/{0.089} \right)/\left[ 3.0\times\left( \sin\theta\right)^{0.8}+0.56 \right]\#\left( 19 \right) \end{aligned}$$

$$\begin{aligned} C=\left\{ \begin{aligned} 1 VFC\leq0.1 \\ 0.6508-0.3436lg{VFC}_{i} 0.1<VFC\leq0.783 \\ 0 VFC>0.783 \end{aligned} \right.\#\left( 20 \right) \end{aligned}$$

$$\begin{aligned} P_{i}=0.2+0.03\alpha\#\left( 21 \right) \end{aligned}$$

$P_{ij}$is the monthly precipitation of grid $\text{i}$, ${SIL}_{i}$ , ${SAN}_{i}$, ${CLA}_{i}$ and ${SOM}_{i}$ stand for silt, sand, clay, and organic matter, $\theta$ means the slope steepness, $\lambda_{i}$ represents the slope length, ${VFC}_{i}$ refers to the vegetation cover fraction, $\alpha$ denotes the percentile slope steepness.

The monetary value of soil conservation comprises the value of reducing sedimentation and retaining soil mineral elements, which is estimated by the replacement cost method and market price method. The equation is as follows:

$$\begin{aligned} V_{S,i}=V_{E,i}+V_{M,i}\#\left( 22 \right) \end{aligned}$$

$$\begin{aligned} V_{E,i}=\left( 24\%\times S_{i}\times{P_{E}}/{\rho_{i}} \right)\#\left( 23 \right) \end{aligned}$$

$$\begin{aligned} V_{M,i}=S_{i}\times ME\times P_{M}\#\left( 24 \right) \end{aligned}$$

$V_{S,i}$represents the monetary value of soil conservation of grid $\text{i}$ (yuan/ha), $V_{E,i}$means the value of reducing sedimentation, $V_{M,i}$ is the value of retaining soil mineral elements. $P_{E}$ represents the cost of reservoir dredging (Jiang et al., 2021), $\rho_{i}$ refers to the soil bulk density of grid $\text{i}$, $ME$ is the content of soil N, P and K, $P_{M}$ refers to the fertilizer price (13).

# Reference

Peng J, Hu X, Qiu S, Hu Y, Meersmans J, Liu Y. Multifunctional landscapes identification and associated development zoning in mountainous area. *Sci. Total Environ.* (2019) 660, 765–775. [doi: 10.1016/j.scitotenv.2019.01.023](https://doi.org/10.1016/j.scitotenv.2019.01.023).

Wang H, Zhou S, Li X, Liu H, Chi D, Xu K. The influence of climate change and human activities on ecosystem service value. *Ecol. Eng.* 87, 224–239. (2016) https://doi.org/10.1016/j.ecoleng.2015.11.027

Wen Y, Sun Q, Yan Y, Xiao M, Song W, Yang J. Impacts of the terrestrial ecosystem changes on the carbon fixation and oxygen release services in the Guangdong-Hong Kong-Macao Greater Bay Area. *Acta Ecol. Sin.* (2020) 40, 8482–8493. [doi: 10.5846/stxb202003310765](http://dx.doi.org/10.5846/stxb202003310765).

Jiang C, Li D, Wang D, Zhang L. Quantification and assessment of changes in ecosystem service in the Three-River Headwaters Region, China as a result of climate variability and land cover change. *Ecol. Indic.* (2016) 66, 199–211. [doi: 10.1016/j.ecolind.2016.01.051](https://doi.org/10.1016/j.ecolind.2016.01.051).

Wang Y, Dai E, Ge Q, Zhang X, Yu C. Spatial heterogeneity of ecosystem services and their trade-offs in the Hengduan Mountain region, Southwest China. *Catena* (2021) 207, 105632. [doi: 10.1016/j.catena.2021.105632](https://doi.org/10.1016/j.catena.2021.105632).

Li M, Liang D, Xia J, Song J, Cheng D, Wu J, Cao Y, Sun H, Li Q. Evaluation of water conservation function of Danjiang River Basin in Qinling Mountains, China based on InVEST model. *J Env. Manage.* (2021) 286, 112212. [doi: 10.1016/j.jenvman.2021.112212](https://doi.org/10.1016/j.jenvman.2021.112212).

An Y, Zhao W, Li C, Sofia SFC. Temporal changes on soil conservation services in large basins across the world. *Catena* (2022) 209, 105793. https://doi.org/10.1016/j.catena.2021.105793.

Benavidez R, Jackson B, Maxwell D, Norton K. A review of the (Revised) Universal Soil Loss Equation ((R)USLE): with a view to increasing its global applicability and improving soil loss estimates. Hydrol. *Earth Syst. Sci.* (2018) 22, 6059–6086. [doi: 10.5194/hess-22-6059-2018](https://doi.org/10.5194/hess-22-6059-2018).

Chen H, Oguchi T, WU P. Assessment for soil loss by using a scheme of alterative sub-models based on the RUSLE in a Karst Basin of Southwest China. *J. Integr. Agric.* (2017) 16, 377–388. [doi: 10.1016/S2095-3119(16)61507-1](https://doi.org/10.1016/S2095-3119(16)61507-1).

Fu B, Liu Y, Lü Y, He C, Zeng Y, Wu B. Assessing the soil erosion control service of ecosystems change in the Loess Plateau of China. *Ecol. Complex.* 8, 284–293. (2011) [doi: 10.1016/j.ecocom.2011.07.003](https://doi.org/10.1016/j.ecocom.2011.07.003).

Liu B, Zhang K, Yun X. An Empirical Soil Loss Equation. Proc. *12th Int. Soil Consevation Organ. Conf.* (2002) 21–25.

McCool DK, Foster GR, Mutchler CK, Meyer LD. Revised Slope Length Factor for the Universal Soil Loss Equation. *Trans. ASABE* 32, 1571–1576. (1989) [doi: 10.13031/2013.31192](https://doi.org/10.13031/2013.31192).

Xu X, Yang G, Tan Y, Liu J, Hu H. Ecosystem services trade-offs and determinants in China’s Yangtze River Economic Belt from 2000 to 2015. *Sci. Total Environ.* (2018) 634, 1601–1614. [doi: 10.1016/j.scitotenv.2018.04.046](https://doi.org/10.1016/j.scitotenv.2018.04.046).

Cui L, Pang B, Li W, Ma M, Sun B, Zhang Y. Evaluation of ecosystem services in the Zhalong wetland. *Acta Ecol. Sin.* (2016) 36, 828–836. [doi: 10.5846/stxb201405161006.](http://dx.doi.org/10.5846/stxb201405161006)
